# Report on hearing loss in oncology

**DOI:** 10.1016/S1808-8694(15)30510-3

**Published:** 2015-10-18

**Authors:** Christiane Schultz, Maria Valéria Schmidt Goffi-Gomez, Patrícia Helena Pecora Liberman, André Lopes Carvalho

**Affiliations:** 1 Master in science, Fundação Antonio Prudente. Speech therapist, Audiology Sector, Hospital do Câncer A C Camargo; 2 Doctor in science of communication disorders, Universidade Federal de São Paulo. Speech therapist, Audiology Sector, Hospital do Câncer A C Camargo; 3 Master in sciences, Fundação Antonio Prudente. Speech therapist, Audiology Sector, Hospital do Câncer A C Camargo; 4 Livre-docente (habilitation) in oncology, FMUSP. Full surgeon, Head & Neck Surgery and Otorhinolaryngology Department, Hospital de Barretos – Hospital do Câncer

**Keywords:** cisplatin/adverse effects, oncology, hearing loss/classification, chemotherapy/adverse effects

## Abstract

Cisplatin is used frequently as an antineoplastic drug in the treatment of many different cancers. However, when used in doses over 360mg/m^2^, ototoxicity may ensue, resulting in loss of hearing. Criteria for identifying and quantifying hearing loss have been devised.

**Aim:**

To describe the features of different hearing loss classification systems and to identify their implications and use in oncologic patients.

**Method:**

Hearing loss was classified in 31 patients before and after chemotherapy, according to different criteria, assessing the sensitivity and specificity of each classification system.

**Results:**

Hearing loss results were highly variable (ranging from 29% to 61%). Only 4 of 31 subjects with post-therapy hearing loss were identified by all the methods. A few subjects with hearing loss were classified as normal hearing in some of the criteria. A normal PTA was found in 18 of 31 subjects in the post-treatment evaluation.

**Conclusion:**

None of the criteria assesses the complaints of patients. The criteria described in this study were inadequate to identify hearing loss following chemotherapy, requiring additional information for physicians to better understand the hearing losses and their implications for the quality of life of patients.

## INTRODUCTION

Cisplatin (CDDP) is an antineoplastic drug used often in the treatment of various tumors. Side effects include: nausea, vomiting, myelosuppression nephrotoxicity, central and peripheral neuropathies, and ototoxicity (Oliveira^1^; Rybak et al.^2^). Ototoxicity may ensue when the drug is given at a cumulative dose over 360mg/m^2^ (Brock et al.^3^; Pedalini et al.^4^, Simon et al.^5^, Knoll et al.^6^). Cisplatin ototoxicity is the result of cochlear injury, initially in the vascular striae and the outer hair cells of the basal gyrus, which result in hearing loss at high frequencies (Rademaker et al.^7^; Rybak et al.^8^). Continued use of the drug may result in hearing loss at low frequencies (Pedalini et al.^4^, Zuur et al.^9^).

Hearing loss may cause significant loss in the quality of life of patients; thus, a concern with ototoxicity should be present throughout oncological therapy. Detecting and monitoring ototoxicity to initiate preventive measures is one of the methods for avoiding hearing loss.

Oncologists are increasingly concerned with drug toxicity in oncological therapy, and have created objective criteria to measure the specific toxicity for each organ during chemotherapy cycles.

Many criteria have been published in the literature, with the aim of identifying, describing and even quantifying hearing loss. In this paper we apply four instruments that will be described below (Davis and Silverman^10^; Brock et al.^3^; ASHA-American Speech-language-Hearing Association^11^; NCI^12^).

In clinical practice, there criteria are difficult to apply, since they do not define clearly the degree of hearing loss or its impact – patient complaints are not taken into account – and do not include all types and grades of hearing alterations.

## OBJECTIVE

The aim of this study was to assess and identify the characteristics of each classification method of hearing loss and to adapt them to oncological monitoring.

## METHOD

A prospective study was proposed and approved by the Research Ethics Committee of the institution in which the investigation was to be conducted (acceptance number 549/03). All patients were consulted about their participation in the study, and signed a free informed consent form. Audiological assessments were carried out in 31 subjects seen at the Audiology unit; inclusion criteria were as follows: cisplatin chemotherapy only; absence of ontological complaints; no radiotherapy in the head and neck, and pre– and post-therapy audiological assessments.

There were 16 male and 15 female patients. Age ranged from 7 to 66 years; the mean age was 28 years.

All subjects in this study underwent full audiological testing before, during and after chemotherapy with CDDP; however, only conventional pure tone audiometry data were computed. A Madsen, Orbiter model version 922 audiometer was used for measuring air and bone conduction pure tone auditory thresholds (in dBHL) (Redondo et al.^13^; Yantis^14^).

Results were tabulated according to classifications proposed by various authors and entities. The CTCAE (Common Terminology Criteria for Adverse Events^12^) was proposed by the NCI (National Cancer Institute). This instrument aims to describe the adverse events due to chemotherapeutic drugs during oncological treatment; numerical values (1 to 4) were given to the reactions observed in each organ during each chemotherapy cycle (Annex 1).

**Annex 1:** Table of the NCI proposed classification Common Terminology Criteria for Adverse Events (CTCAE) (2003) Cancer Therapy Evaluation Program, Common Terminology Criteria for Adverse Events Version 3.0 DCTD, NCI, NIH, DHHS. March31, 2003 (http://ctep.cancer.gov) Publishing date, 10 June 2003

Hearing / Ear						
Auditory Adverse Event	Name	1	2	3	4	5
patient with or with no baseline audiogram and non-monitored patients	Hearing: (monitoring program)	Thresholds or 15 – 25dB decrease relative to the baseline audiogram at two or more adjacent frequencies in at least one ear, or subjective change in the absence of grade 1	Thresholds or 25 in two or more adjacent frequencies in at least one ear	ADULTS: thresholds over 25 – 90 dB at 3 adjacent frequencies in at least one ear.	ADULTS: profound bilateral hearing loss (> 90dB)	
				CHILDREN: Hearing loss sufficient to require treatment including AASI (loss > 20dB at speech frequencies, bilateral or > 30dB unilateral and that require special phonoaudiological care)	CHILDREN: Audiological indication for cochlear implants, requiring special phonoaudiological care.	–
OBS: pediatric recommendations are similar to those of adults unless otherwise specified. For children and teenagers (< or equals; 18 years) with no pre-treatment baseline audiogram, hearing is considered as below 5dB.						
Hearing: patients with no baseline audiogram and not included in a monitoring program	Hearing: no monitoring program		Hearing loss with no need to use AASI (no effect on activities of daily life)	Hearing loss with no deed to use AASI (affecting activities of daily life)	Profound bilateral hearing loss (> 90dB)	–
Otitis, outer ear (non– infectious)	Otitis media	Otitis external with erythema or dry desquamation	Otitis external with desquamation, ear wax or effusion, tympanic perforation, tympanoplasty	Otitis external with mastoiditis, stenosis or osteomyelitis	Necrosis of bone or soft tissues	Death
OBS: patients with or with no baseline audiogram, included or not in a monitoring program.						
Otitis, middle ear (non– infectious)	Otitis media	Otitis serous	Otitis serous with indication for medical intervention	Otitis with effusion, mastoiditis	Necrosis of canal, of bone or soft tissues	Death
Tinnitus	Tinnitus	–	Tinnitus not affecting activities of daily life	Tinnitus affecting activities of daily life	Disability Loss of function	–
OBS: patients with or with no baseline audiogram, included or not in a monitoring program.						
Hearing / Ear Others	Hearing / Ear Others	mild	moderate	severe	life risk / loss of function	Death

Brock et al.^3^ attributed number values (0 to 4) to various types of hearing loss due to chemotherapy (Annex 2).

**Annex 2:** Descriptive table of Brock et al.'s proposed classification (1991)

Hearing loss Bilateral	Grade
< 40dB at all frequencies	0
≥ 40 dB only at 8.000Hz	1
≥ 40 dB starting at 4.000Hz	2
≥ 40 dB starting at 2.000Hz	3
≥ 40 dB starting at 1.000Hz	4

The ASHA^11^ considers ototoxicity as a 20dB threshold elevation at a specific frequency, a 10dB threshold elevation at two consecutive frequencies, or absent responses at three consecutive frequencies in post-therapy testing.

Davis and Silverman^10^ classify the degree of hearing loss according to the mean value of thresholds at 500, 1000 and 2000Hz; normal values – 0 to 20 dB, mild hearing loss – 21 to 40 dBHL, moderate hearing loss – 41 to 70 dBHL, severe hearing loss – 71 to 90 dBHL, profound hearing loss - over 95 dBHL.

A comparison was done taking the 25 dBHL threshold as the limit between normal hearing and hearing loss to assess the sensitivity and specificity of each criterion, with the aim of analyzing hearing loss according to frequency (PPF). Fisher's exact test was applied to study the significance of each method. The GraphPad Prism version 2.0 software was used. The significance level was < 0.05.

## RESULTS

Table 1 shows that, according to NCI12 criteria, 12 of 31 patients (38%) presented hearing loss at the end of therapy. Of these, only one had grade I changes, 10 had grade II changes, and one had grade III changes. According to Brock et al.'s^3^ criterion, 19 patients (61%) presented hearing loss at the end of therapy. Of these, 5 had grade I changes, 5 had grade II changes, 5 had grade III changes, and 4 had grade IV changes. According to ASHA^11^ criteria, 17 subjects (54%) presented hearing loss at the end of therapy. Of these, 13 had a 10dB loss in two or more frequencies, and 4 had a 15dB loss or more in only one frequency. However, 39% of patients had a loss over 15dB in two or more consecutive frequencies in at least one ear.

**Table 1:** Distribution of hearing loss classifications in the study population according to the criteria applied.

Patient	BROCK	ASHA (2002)	NCI	SILVERMAN	PPF
1	0	0	0	Normal	Normal
2	0	0	0	Normal	Normal
3	0	0	0	Normal	Normal
4	0	0	0	Normal	Normal
5	0	0	0	Normal	Normal
6	0	0	0	Normal	Normal
7	0	0	0	Normal	Normal
8	0	1	0	Normal	No
9	1	0	0	Normal	No
10	0	1	0	Normal	8
11	1	1	2	Normal	8
12	0	0	0	Normal	6
13	0	0	0	Normal	6
14	0	1	0	Normal	250,500
15	1	1	0	Mild	250,8
16	2	0	0	Normal	4,6,8
17	3	0	2	Mild	6,8
18	2	1	2	Normal	4,6,8
19	2	1	2	Normal	4,6,8
20	2	1	2	Normal	4,6,8
21	3	1	0	Normal	4,6,8
22	3	1	2	Normal	4,6,8
23	3	1	2	Normal	4,6,8
24	3	1	2	Normal	4,6,8
25	1	1	0	Mild	2.4.6.8
26	4	1	2	Mild	2,3,4,6,8
27	2	0	0	Mild	All
28	1	1	1	Mild	All
29	4	1	2	Mild	All
30	4	0	0	Moderate	All
31	4	1	3	Moderate	All

Davis and Silverman's^10^ criteria revealed that 9 subjects (29%) had hearing loss at the end of therapy. The identification percentage of hearing loss varied considerably among criteria - from 29% to 61%. We also found that 61% had auditory thresholds over 25 dBHL in at least one frequency bilaterally.

Statistical analysis consisted of specificity and sensitivity calculations for each criterion; Fisher's test was applied to calculate the p value, as shown on the Table below.

Table 2 shows Brock's (1991)^3^ proposed criteria; it had the highest sensitivity. The NCI's^12^ and Davis and Silverman's^10^ criteria were the most specific.

**Table 2:** Sensitivity and Specificity, with their respective confidence intervals (CI), for each criterion.

Criteria				Hearing loss	
			YES	NO	
Brock	Hearing loss	YES	18	1	Specificity: 81.8%
		NO	4	8	Sensitivity: 88.9%
ASHA (2002)	Hearing loss	YES	16	1	Specificity: 88.9%
		NO	6	8	Sensitivity: 72.7%
NCI	Hearing loss	YES	12	0	Specificity: 100%
		NO	10	9	Sensitivity: 54.5%
SILVERMAN	Hearing loss	YES	9	0	Specificity: 100%
		NO	13	9	Sensitivity: 40.9%

## DISCUSSION

The distribution of audiometric thresholds in the study sample showed that:

• Only 4 subjects with post-therapy hearing loss were identified by all criteria; 7 subjects had no hearing loss in any of the criteria applied in this study.
• Thirteen of 18 subjects with normal pure tone audiometry (according to Davis and Silverman^10^) after therapy had some degree of hearing loss according to the other methods applied in this study.

Brock et al.'s^3^ criteria demonstrated the highest number of significant hearing losses; the ASHA^11^ criteria only revealed whether there was hearing loss or not, but provided no quantification.

Auditory losses due to cisplatin ototoxicity are generally symmetrical, bilateral, initially affecting high frequencies, followed by middle and low frequencies (Testa et al.^15^; Rademaker et al.^7^).

Changes were not always detected at the beginning by the criteria described above. A major point about these criteria is that they do not take the complaints of patients into account, which would be extremely important. Additionally, the impact on a patient's life may not be proportional to the degree of hearing loss, since this impact depends on factors such as social and professional activities and personal aspects. The growing concern on the part of oncologists should lead to careful prevention of hearing loss, comprising periodic testing and close monitoring of hearing losses, not waiting for patients to complain before becoming concerned with hearing; this means preventing the onset of hearing loss and not only rehabilitation when hearing cannot be recovered any longer.

A relevant point was that NCI^12^ criteria were not sufficiently specific for monitoring auditory function; it does not define clearly which frequencies should be investigated.

Audiologically, there are monitoring proposals using conventional frequencies (Testa et al.^15^, Kushner et al.^16^, Marshall et al.^17^, Toral-Martinnon et al.^18^), monitoring proposals using transient otoacoustic emissions (Liberman^19^) or distortion products (Biro et al.^20^; Hyppolito et al.^21^), and monitoring proposals using high frequencies (over 8000 Hz) (Garcia^22^), which may be first affects by ototoxic drugs. In this case, patients with hearing loss normally do not complain and not always perceive loss of hearing (Liberman^23^). Dhooge et al.,^24^ however, found symptomatic ototoxicity in 20% of cases - auditory complaints in 16 children treated with cisplatin and/or carboplatin. Monitoring with conventional frequencies (500 to 8000 Hz) shows that hearing loss leads to difficulties in different situations; patients may complain only of tinnitus or difficulties to understand speech in noisy environments, or may present hearing loss at speech frequencies, no longer being able to follow a conversation. Still in the criterion for patients with auditory changes in pre-therapy testing, these subjects may be classified as not having post-therapy auditory changes; but therapy will have caused elevated audiometric thresholds, which are not taken into account in the final criterion.

A further discrepancy is the grade II in this criterion. This grade includes patients that had a 25 to 90 dB elevation of the auditory threshold. A 25 dB threshold elevation may go unnoticed or may cause minimal difficulty; it may even be classified as mild hearing loss (Davis and Silverman^10^). On the other hand, a 90 dB elevation suggests that subjects will probably not follow a conversation without using hearing aids, since voice during a conversation is issued at around 60 dBHL. Furthermore, hearing loss here is classified as severe, which brings significant restrictions on social life. Classifying these two extremes of threshold elevations in the same grade does not take into account the significant differences, complaints and limitations.

The 40 dB reference in Brock et al.'s^10^ proposed criteria is debatable. A 40 dB loss characterizes mild hearing loss, with serious implications for the perception of Portuguese consonants (Russo and Behlau^25^), especially in children, for which this classification was proposed. This author does not take into account changes above 40 dB that may occur at single frequencies, as well as not considering small auditory alterations that may occur before the auditory threshold reaches 40 dB. Intensity is an important factor in hearing loss; it is also important in rehabilitation with hearing aids, and may be a limiting factor in choosing and using an appropriate aid.

The ASHA^11^ criteria do not account for affected frequencies; from an audiological perspective this has implications for the follow-up of oncological therapy. A 10 dB threshold increase at 6000 and 8000 Hz may not result in minimal hearing loss, depending on age, while a 20 dB decrease at 1000 and 2000 Hz in a patient with a 30 dB pre-treatment threshold causes moderate hearing loss and may result in significant communication difficulties. Thus, the affected frequency should be taken into account.

Davis and Silverman^10^ proposed criteria is not indicated for oncological patients, since it classifies hearing loss only at 500, 1000 and 2000 Hz thresholds, which are not those commonly involved in ototoxicity.^1^,^26^, ^27^ This is a problem, because when ototoxic drug induced hearing loss affects these frequencies, loss is already significant and patients present major complaints; it is thus inadequate for monitoring patients at risk of hearing loss - when this criterion detects loss, it is already rather advanced.

The same subject with hearing loss was not always classified by all of the criteria above; thus, the sensitivity and/or specificity of each criterion needs to be known, as small changes in hearing are not always detected by these instruments. Important hearing losses are easily detected by any of these tools; however, a classification instrument should detect small changes so that oncologists and speech therapists may carefully monitor the hearing function of these patients, to avoid major loss of function.

The classification of hearing loss in audiological evaluations during oncological therapy aims firstly to identify ototoxic effects, especially at high frequencies. This makes it possible for physicians to be alert and change therapy protocol measures. Secondly, the classification should indicate at which point patients will suffer the social, educational and professional implications of hearing loss. Impact differs in adults and children, both in terms of the degree of hearing loss and the affected frequencies. A classification should be able to show the progression of hearing loss.

Knight et al.^28^ monitored ototoxicity in 67 children with osteosarcoma, neuroblastoma and medulloblastoma, all of which were treated with cisplatin. These authors compared the Brock et al.,^3^ ASHA,^11^ and NCI^12^ criteria, and also found it difficult to adequately describe hearing losses. These authors believe that those criteria underestimate hearing loss resulting from oncological therapy; the result of this is that language development, learning and social/emotional function may be compromised in these children. These authors also found that hearing loss may lead to low self-esteem, behavioral disorders, loss of energy and stress, compared to normal hearing children; these factors are not included in any of these classification systems.

Liberman^29^ studied patients with cancer treated during childhood with cisplatin, and found a higher occurrence of auditory complaints when hearing loss affected the 4000Hz frequency.

Marini et al.^30^ analyzed the predictive power, sensitivity and specificity of auditory complaints in 795 patients and found that the sensitivity was high (80.9%) and the specificity was 60.4%. Audiometric test results should also be available; although more subjective, these results are less costly than new technologies.

Teles et al.^31^ compared data such as frequency, proportion, agreement and consistency of responses in workers exposed to occupational noise; significant changes were noted in the audibility threshold (MSL). These authors applied three Brazilian criteria and one international criterion to analyze threshold changes and found that these criteria in themselves were inadequate, given their subjective nature; prevention would be necessary not only in subjects presenting MSL but in all subjects in an auditory preservation program.

Gupta et al.^32^ found a small incidence of hearing loss in children undergoing cisplatin chemotherapy by continuous infusion; these authors used Brock's criterion and concluded that continuous drug administration is associated with a lower incidence of ototoxicity. However, we believe that this criterion underestimates important losses in this population because it takes into account only losses over 40 dB.

According to the literature we cited, changes in hearing were not always detected at the beginning in oncological patients by the criteria described in this study. The main criticism of these criteria is that they do not include patient complaints, essential for understanding the impact of hearing loss on patient's lives, which are not always proportional do the degree of loss, but depend on factors such as personal, social and professional activities. The growing concern of oncologists should lead to preventive care to avoid hearing loss; this includes periodic testing and close monitoring of auditory changes before patients complain to avoid hearing loss rather than just rehabilitation when reversion is no longer possible.

Each classification tool should be assessed according to its purpose. In the case of hearing loss associated with oncological therapy, we believe it is relevant not only to identify, classify and quantify auditory losses, but also to investigate eventual complains and the impact that hearing loss may cause on the quality of life of patients. It is impossible to define the degree of hearing loss that results in complaints and difficulties for patients using only the criteria mentioned above for classifying auditory losses. Thus, identifying and validating the complaints of patients is just as important as audiological evaluations and classification of losses according to charts.

It is extremely important to note that the criterion used for classifying hearing losses in oncological patients should be able to identify the beginning of loss, thus avoiding unnecessary adverse effects and preventing auditory losses due to oncological therapy.

## CONCLUSION

We found that Davis and Silverman's^10^ criteria showed hearing loss in 29% of subjects at the end of treatment; the NCI^12^ criteria showed hearing loss in 38% of patients; the ASHA^11^ criteria showed hearing loss in 54%; and Brock et al.'s^3^ criteria showed hearing loss in 61% of patients at the end of oncological therapy. Thus, Brock's (1991)^3^ proposed criteria was the most sensitive, and the NCI's^12^ and Davis and Silverman's^10^ criteria were the most specific.

However, all of these criteria underestimated the description of identified auditory alterations; additional information was required to help physicians to understand the true implications of hearing loss in each case.

A common code is needed between audiologists and oncologists to increase our understanding and improve the therapy of these patients; not only should the grade and type of hearing loss be described, but also the impact on the lives of patients.
